# Distinct heat response molecular mechanisms emerge in cassava vasculature compared to leaf mesophyll tissue under high temperature stress

**DOI:** 10.3389/fpls.2023.1281436

**Published:** 2023-11-30

**Authors:** Shujuan Wang, Xincheng Zhou, Kun Pan, Huaifang Zhang, Xu Shen, Jia Luo, Yuanchao Li, Yinhua Chen, Wenquan Wang

**Affiliations:** ^1^ College of Tropical Crops, Hainan University, Haikou, Hainan, China; ^2^ Institute of Tropical Biosciences and Biotechnology, Chinese Academy of Tropical Agricultural Sciences (CATAS), Haikou, China; ^3^ Hainan Provincial Key Laboratory for Research and Development of Tropical Herbs, Haikou Key Laboratory of Li Nationality Medicine, Hainan Ouality Monitoring and Technology Service Center for Chinese Materia MedicaRaw Materials, School of Pharmacy, Hainan Medical University, Haikou, Hainan, China

**Keywords:** high temperature, transcriptome, leaf, mid-veins, vascular bundle

## Abstract

With growing concerns over global warming, cultivating heat-tolerant crops has become paramount to prepare for the anticipated warmer climate. Cassava (*Manihot esculenta Crantz*), a vital tropical crop, demonstrates exceptional growth and productivity under high-temperature (HT) conditions. Yet, studies elucidating HT resistance mechanisms in cassava, particularly within vascular tissues, are rare. We dissected the leaf mid-vein from leaf, and did the comparative transcriptome profiling between mid-vein and leaf to figure out the cassava vasculature HT resistance molecular mechanism. Anatomical microscopy revealed that cassava leaf veins predominantly consisted of vasculature. A thermal imaging analysis indicated that cassava experienced elevated temperatures, coinciding with a reduction in photosynthesis. Transcriptome sequencing produced clean reads in total of 89.17G. Using Venn enrichment, there were 65 differentially expressed genes (DEGs) and 93 DEGs had been found highly specifically expressed in leaf and mid-vein. Further investigation disclosed that leaves enhanced pyruvate synthesis as a strategy to withstand high temperatures, while mid-veins fortified themselves by bolstering lignin synthesis by comprehensive GO and KEGG analysis of DEGs. The identified genes in these metabolic pathways were corroborated through quantity PCR (QPCR), with results aligning with the transcriptomic data. To verify the expression localization of DEGs, we used *in situ* hybridization experiments to identify the expression of *MeCCoAMT(caffeoyl-coenzyme A-3-O-methyltransferase*) in the lignin synthesis pathway in cassava leaf veins xylem. These findings unravel the disparate thermotolerance mechanisms exhibited by cassava leaves and mid-veins, offering insights that could potentially inform strategies for enhancing thermotolerance in other crops.

## Introduction

High-temperature (HT) stress poses a significant challenge to global crop production and food security, exacerbated by the escalating impacts of global warming. Crop species such as soybean, corn, and rice have exhibited reduced yields under HT stress (Lobell and Asner, 2003; Peng et al., 2004). This crisis is becoming aggravated due to the global warming (Bita and Gerats, 2013). This crisis underscores the urgency to comprehend the intricate impacts of HT stress on plant growth and production, spanning physiology, biochemistry, and gene regulation. Physiologically, HT stress induces scorching, accelerates leaf senescence, and hampers shoot and root growth (Vollenweider and Gunthardt-Goerg, 2005). Furthermore, HT stress impairs crop production by diminishing shoot net assimilation rates (Wahid et al., 2007). Biochemically, HT stress triggers metabolic shifts, with the emergence of hydrogen peroxide (H_2_O_2_) damaging plant cell lipid membranes (Hasanuzzaman et al., 2013). And prolonged warming inhibited the tricarboxylic acid pathway in Arabidopsis (Wang et al., 2020). Biochemically, HT stress triggers metabolic shifts, with the emergence of H_2_O_2_ damaging plant cell lipid membranes (Krasensky and Jonak, 2012).

The effects of observed heat stress are contingent not only upon distinct biological levels but also specific plant organs. In whole wheat seedlings, HT stress reduces the chlorophyll content and membrane stability index, while increasing proline content (Gupta et al., 2013). In leaves, photosynthesis rates decrease in mature and dependent leaves; concurrently, HT stress curtails stomatal conductance in *Coffea arabica *(Marias et al., 2017). In roots, short-term severe heat stress diminishes total protein concentration, along with levels of nutrient uptake and assimilation proteins in tomatoes (Giri et al., 2017). The vascular system also experiences alterations, as HT stress diminishes xylem vessel density in *Phragmites australis*. Notably, research concerning HT stress’s impact on the vascular system remains scarce compared to other plant tissues. More importantly, vascular bundles play a crucial role in the plant’s resistance processes, such as transmitting signaling molecules and maintaining the resilience of plant tissues, among other functions.

The plant vascular system plays a pivotal role in resource distribution across plant organs and offers mechanical support. Additionally, it serves as a communication nexus between shoots and roots (Lucas et al., 2013). Vascular system development is influenced by temperature fluctuations (Wei et al., 2023). Therefore, the vasculature system has evolved many different bio-progresses for plant to adapt to the unfavorite environment cues. Analyzing the composition of phloem and xylem sap in stressed plants is a popular approach to studying vascular responses to abiotic stress (Hu et al., 2016). For example, there were 2558 proteins had been identified in tomato phloem sap under the water deficiency condition (Ogden et al., 2020).

Research on vascular responses to abiotic stress predominantly employs vascular sap as the study subject, facilitated by EDTA. However, this approach is marred by issues of contamination linked to both EDTA facilitated method and cucurbits exudation (Zhang et al., 2012). While insect stylets offer relatively pure phloem sap devoid of contamination, their limited sap exudate volume restricts their utility (Reidel et al., 2009). Furthermore, these methods exclusively identify mobile molecules, potentially overlooking the roles of immobile molecules synthesized in the phloem as stress responses (Sevanto, 2014). For example, researcher found that vascular bundle could mediate systemic reactive oxygen signaling during light stress (Zandalinas et al., 2020). Furthermore, these methods exclusively identify mobile molecules, potentially overlooking the roles of immobile molecules synthesized in the phloem as stress responses.

Cassava (*Manihot esculenta Crantz*) stands out as a vital tropical crop due to its robust root productivity and remarkable HT tolerance (Wang W. et al., 2014). As a tropical crop, cassava deploys a variety of adaptive, avoidance, and acclimation strategies to counter HT stress. However, precisely because it is a tropical crop, its heat tolerance is often considered the norm, leading to a paucity of research on its specific heat-resistant capabilities. This is especially true for studies focusing on leaf tissue and vascular bundles. Therefore, comprehending cassava’s responses to HT stress in its vasculature and leaf mesophyll holds significance for enhancing heat tolerance in other crops.

## Materials and methods

### Growth condition and heat treatment

We used Cassava variety KU50 as materials in this study. When KU50 seedlings sprouted, we only let one seedling in one pot. Then we kept plants continuously growing in greenhouse with the day/night temperature at 35/28°C. We initiated high temperature (HT) treatment for KU50 at approximately 45 days after planting by increasing the air temperature to 45°C for three hours. And the control set of KU50 was watered normally and grew in the same growth condition in normal temperature.

### Physiological measurements

We observed the cassava leaf vein by using the light microscope before the treatment to make sure the tissue collection was right for this study. We used the Toluidine Blue O to stain the Cassava leaf vein and observed by using the light microscope. Then cassava KU50 seedlings were treated with high temperature (HT) by increasing the air temperature from 35°C to 45°C for three hours. During the HT treatment, we used the “Flir-One” system (FLIR, Nashua, NH) to measure the leaf temperature on three leaves localized on top part of the cassava seedlings. After the treatment, we used a Li-Cor 6800 (LI-COR, Lincoln, NE) photosynthesis apparatus to measure net photosynthetic rate, transpiration, and stomatal conductance on the top three fully expanded leaves following the instruction from the manufacturer.

### Statistical analyses

To compare the rates for photosynthesis, water potential, stomatal conductance, six biological replicates and three technical replicates were used for each of these measurements. The results were mean ± SE of these independent replicates.

### RNA extraction and RNA-Seq library construction

We collected three top cassava leaves from each of the control and heat stressed KU50 and then put them together as one sample and one bio-replicate, we took 3 bio-replicates for each treatment. Then we put all samples into liquid nitrogen. Total RNA was extracted by E.Z.N.A plant RNA kit (Omega Bio-Tek Company, Norcross, GA). We monitored RNA degradation and contamination by using 1% agarose gels. And RNA purity was assessed using the NanoDrop spectrophotometer (NanoDrop Technologies Inc., Wilmington, DE). And we checked RNA integrity by using the RNA Nano 6000 Assay Kit of the Bioanalyzer 2100 system (Agilent Technologies, CA, USA). A total amount of 1 μg RNA per sample was used as input material for the RNA sample preparations. Sequencing libraries were generated using NEBNext^®^ UltraTM RNA Library Prep Kit for Illumina^®^ (NEB, USA) following manufacturer’s recommendations and index codes were added to attribute sequences to each sample.

### Transcriptomic data analysis

The library preparations were sequenced on an Illumina Novaseq platform and 150 bp paired-end reads were generated. And Raw data (raw reads) of fastq format were processed through in-house perl scripts, then clean data (clean reads) were obtained by removing reads containing adapter, reads containing ploy-N and low-quality reads from raw data. At the same time, Q20, Q30 and GC content the clean data were calculated. Reference genome and gene model annotation files were downloaded from genome website directly. Index of the reference genome was built using Hisat2 v2.0.5 and paired-end clean reads were aligned to the reference genome using Hisat2 v2.0.5. And then FPKM of each gene was calculated based on the length of the gene and reads count mapped to this gene. Differential expression analysis of heat treatment and control for KU50 was performed using the DESeq2 R package. Genes with an adjusted P-value <0.05 found by DESeq2 were assigned as differentially expressed.

Gene Ontology (GO) enrichment analysis of differentially expressed genes was implemented by the clusterProfiler R package, in which gene length bias was corrected. GO terms with corrected P-value less than 0.05 were considered significantly enriched by differential expressed genes. KEGG is a database resource for understanding high-level functions and utilities of the biological system, such as the cell, the organism and the ecosystem, from molecular-level information, especially large-scale molecular datasets generated by genome sequencing and other high-through put experimental technologies (http://www.genome.jp/kegg/). We used clusterProfiler R package to test the statistical enrichment of differential expression genes in KEGG pathways.

### RNA preparation, reverse transcription, and quantitative polymerase chain reaction

Total RNA was extracted from tissues using RNAprep Pure Plant kit (TIAGEN, Beijing, China). RNA amounts were estimated using a NanoDrop 2000 (Thermo FisherScientific, Waltham, MA USA). cDNA synthesis was performed with approximately 2 µg of total RNA and the reaction system followed the HiFi II M-MLV (H-) Reverse Transcriptase Kit with gDNA Eraser (CWBIO, CW07435, China) protocol. The cDNA was eluted with 100 µL deionized water. A total of 2 µL of eluted cDNA was used as a template for QPCR analysis. QPCR was performed on a BioRad sequence detection system using MonAmp™ SYBR^®^ Green qPCR Mix (Low ROX) (Monad, RR820, MQ10201S, China) for detection. Amplification conditions were as follows: 3 min of initial denaturation at 95°C, followed by 40 cycles of 30 s at 95°C, 30 s at 56°C, and 30 s at 72°C. Melt curves used to determine primer specificity were 10 s at 95°C, followed by cycles of 0.05 s at 65–95°C, rising 0.5°C/cycle. Primers are listed in **Supplemental Table 1**. And the relative expression level was calculated by 2^−ΔΔct^ method. Each include three technical replicates. The FPKM values of DEGs in transcriptome data can be used as a reference for their expression patterns.

### In tube *in situ* PCR on tissue sections of cassava leaf mid-vein

In tube *in situ* PCR was carried out on sections of unfolded leaves, and midveins from the fully unfolded leaf from 2-month-old cassava plants of KU50 as described previously using primers 5’-ATGATTAATCTTGTGTATTATTTGGTGA-3’ and 5’-TCAGGAAATGCGCCTGCAGAT-3’ for *MeCCoAMT*. The specificity of the primers was verified by DNA sequencing of the PCR products. Prior to sectioning, all tissues were fixed in FAA (2% formaldehyde, 5% acetic acid, and 60% ethanol in phosphate buffered saline [PBS]) for 2h at 4°C and then washed three times with washing buffer (60% ethanol and 5% acetic acid in PBS). The tissue was embedded in 5% agarose in PBS to enable sectioning into 80 um tissue sections on a Leica vibratome. The sections were treated 4h with 30µl of RNase-free DNase (40u) and RNase inhibitor (4U) at 37°C and subsequently washed twice with water. To partly release the coagulation of the fixative, the sections were incubated in 30 µl of Pepsin (2ng in 1µl 10mM HCl) for 15 min prior to the reverse transcriptase reaction, and the sections were washed twice in water. The mRNA was translated to cDNA using the specific reverse primers for *MeCCoAOMT* using the protocol from Sensiscript (Qiagen 1017746). Before the cDNA was amplified, the reverse transcriptase solution was removed and the PCR reagents were added. DIG-labeled dUTP was incorporated as the basis for visualization. The expression was subsequently visualized using e PCR ELISA, DIG-Detection package (Roche Cat. No. 11 965 409 910). The more details see the manual protocol of PCR ELISA, DIG-Detection package (Roche Cat. No. 11 965 409 910). The sections were mounted on glass slides and examined using a Leica DM microscope.

## Results

### Effects of HT stress on the cassava KU50 and the tissue sampling

**Figure 1:**
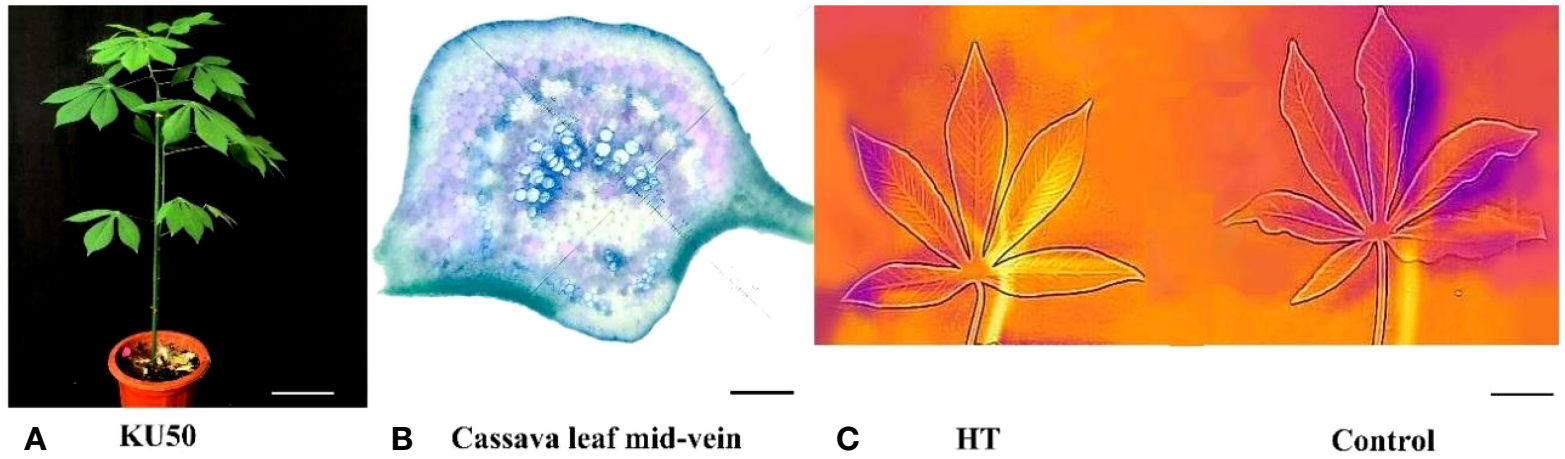
Phenotype of plant KU50 under HT **(A)**. Normal plant of KU50 (Bar means 9cm) **(B)**. The transection of KU50 leaf vein stained by toluidine blue O (Bar means 0.2mm) **(C)**. The thermal imaging figure of leaves for the HT KU50 and Control (Bar means 3cm).

Emerging evidence highlights the divergent response mechanisms of distinct plant tissues to abiotic stress. To elucidate the molecular reaction of cassava vasculature to HT stress, we conducted a comparative transcriptome analysis using cassava leaf and mid-vein samples. Prior to HT treatment, we examined the physiological structure of cassava leaf mid-veins through Toluidine Blue staining and microscopy. The chosen cassava KU50 seedlings (**Figure 1A**) possessed a mature root system, making them suitable for HT stress assessment. Toluidine Blue-stained cross sections of the main veins (**Figure 1B**) displayed a clear vasculature structure, with deep, green-stained xylem surrounded by phloem, enabling precise dissection of the mid-vein vascular bundle from the leaf. However, it’s essential to acknowledge that this method might retain some mesophyll tissue around the vasculature due to incomplete separation. Consequently, we employed comparative analysis of heat-stressed leaf and mid-vein samples to unravel the molecular mechanisms of vasculature’s response to heat stress.

During HT treatment, we employed the “Flir One” system to measure leaf temperature and Li-Cor 6800 to gauge photosynthetic parameters. The thermal image of cassava leaves revealed a shift from orange and blue (low temperature) to light yellow and orange (high temperature) under HT treatment, indicating elevated leaf temperatures due to heat stress (**Figure 1C**). The observed color change reinforced the notion that KU50 was indeed subjected to HT stress.

**Figure 2:**
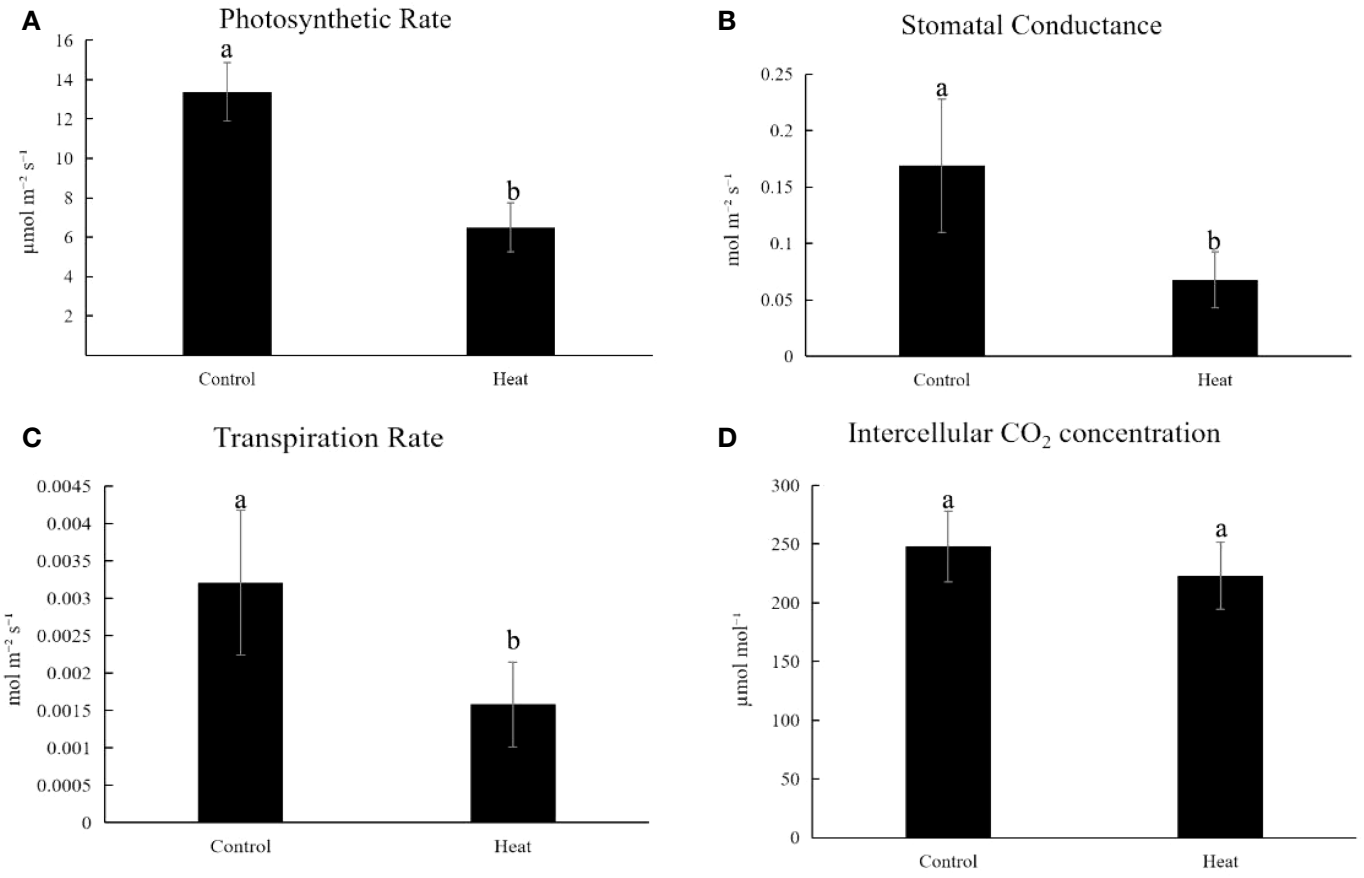
Changes of physiological parameters related to photosynthesis of plant KU50 under HT stress **(A)**. The photosynthetic rate for HT treated cassava **(B)**. Stomatal Conductance for HT treated cassava **(C)**. Transpiration rate for HT treated cassava **(D)**. Intercellular CO_2_ Concentration for HT treated cassava. Lowercase letters are difference significance markers.

Compared with control, HT-stressed cassava exhibited a significant reduction in net photosynthetic rate, declining from 13.38μmolm^-2^s^-1^ to 6.50μmolm^-2^s^-1^ (**Figure 2A**). Concurrently, stomatal conductance decreased from 0.1689molm^-2^s^-1^ to 0.06787molm^-2^s^-1^ (**Figure 2B**), while transpiration rate dropped from 0.0032molm^-2^s^-1^ to 0.00158molm^-2^s^-1^ (**Figure 2C**). Furthermore, the intercellular CO_2_ concentration decreased from 248.201μmolm^-2^s^-1^ to 223.04μmolm^-2^s^-1^ under HT stress (**Figure 2D**).

### Transcriptome analysis revealed the different molecular responses from vasculature to leaf mesophyll in cassava under HT stress

#### Transcripts annotated response to HT stress

**Figure 3:**
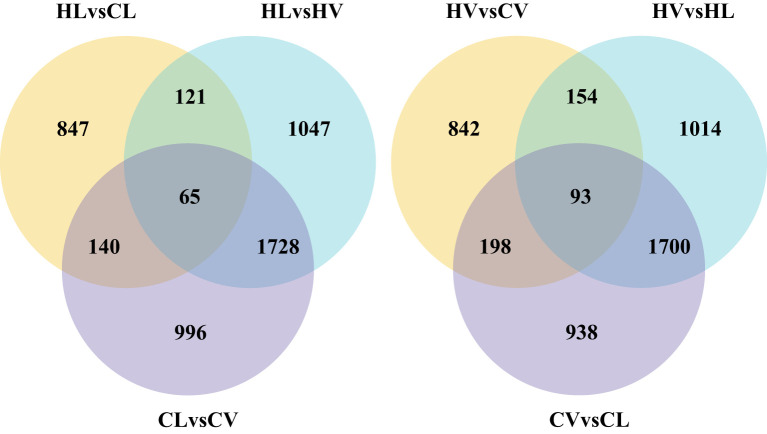
Venn picture for DEGs of leaf vasculature and mesophyll under HT stress compared with normal condition (HL, HT stressed leaf mesophyll; HV, HT stressed leaf vein; CL, control leaf mesophyll; CV, control leaf vein).

For this study, we collected three sets of HT-stressed leaf mesophyll samples, three sets of HT-stressed mid-vein samples (HL and HV), and three control samples each of control leaf (CL) and mid-vein (CV) for RNA-Seq analysis. The assembly and annotation outcomes are summarized in **Table 1**. In total, we generated 613.94 million raw reads, of which 594.47 million were deemed clean reads. While manually dissecting the mid-vein from the leaf is not entirely precise in segregating vasculature from leaf mesophyll, we took steps to enhance the identification of tissue-specific differentially expressed genes (DEGs) responding to HT stress. Our data underwent a Venn diagram-based filtering approach (Log2 fold change > 2, p-value < 0.05) to focus on significant differences. Specifically, we compared the transcripts between KU50 leaf mesophyll and mid-vein under high-temperature treatment against their respective control sets (**Figure 3**).

**Table 1:** Summary of RNA-Seq data of KU50 under HT stress and the control.

Sample	Raw Reads	Clean Reads	Error Rate	Q20	Q30	GC content (%)
HL1	62473626	61225014	0.03	97.94	93.91	44.86
HL2	44824028	44090450	0.03	97.8	93.6	43.27
HL3	54217986	52275694	0.03	97.81	93.6	43.23
HV1	49484736	47971474	0.03	97.86	93.71	44.43
HV2	45441586	44077656	0.03	97.63	93.19	43.97
HV3	53481614	52269222	0.03	97.86	93.72	43.29
CL1	54116060	51915060	0.03	97.95	93.95	44.41
CL2	49519458	47648818	0.03	97.94	93.9	45.28
CL3	53559728	51311908	0.03	97.96	93.96	45.18
CV1	42036088	40568510	0.03	97.88	93.79	44.69
CV2	49488362	47763428	0.03	97.83	93.64	44.48
CV3	55292162	53348210	0.03	97.9	93.83	44.51
Total	613935434	594465444				

The analysis yielded 1287 DEGs in the mid-vein and 1173 DEGs in the mesophyll tissue in response to HT stress. Remarkably, we identified 93 DEGs enriched within the HT-stressed mid-vein vasculature and 65 DEGs enriched in the mesophyll tissue under HT treatment. These findings underscore that cassava’s mid-vein vasculature exhibits a more pronounced DEG response than the leaf under high-temperature stress.

#### Mid-vein vasculature had different HT responsive mechanism compared with leaf mesophyll

**Figure 4:**
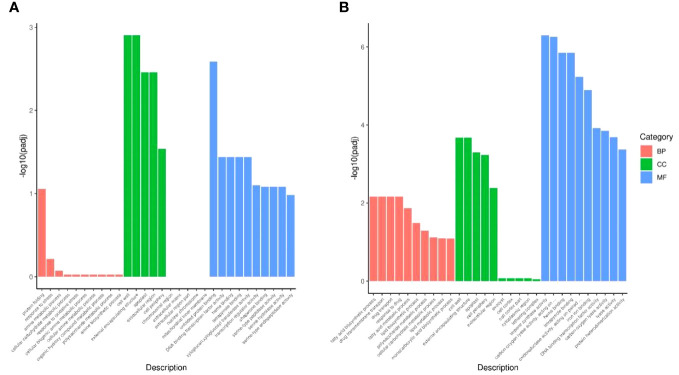
GO term for HL **(A)** and HV **(B)**.

To comprehensively assess the functional implications of the DEGs of HV and HL, GO analysis was performed and all DEGs were classified into three categories: biological process, cellular component and molecular function. Remarkably, our investigation revealed that mid-vein vasculature displayed a more pronounced array of heat-responsive molecular activities compared to leaf mesophyll (**Figure 4**). In the realm of biological processes, DEGs in HL were grouped into “protein folding,” “response to stress,” and “amine metabolic process,” suggesting leaf involvement in reactive oxygen species (ROS) reactions. Notably, the cellular component analysis showcased associations with “cell wall,” “external encapsulating structure,” “apoplast,” “extracellular region,” and “cell periphery,” indicative of leaf engagement in sucrose dynamics and cell osmotic regulation. Furthermore, molecular function analyses linked DEGs to “unfolded protein binding,” “DNA binding transcription factor activity,” “heme binding,” “tetrapyrrole binding,” and “xyloglucan:xyloglucosyl transferase activity,” underscoring leaf participation in photosynthesis and chlorophyll synthesis. Contrastingly, DEGs in HV were allocated to “fatty acid biosynthetic process,” “drug transmembrane transport,” “drug transport,” “response to drug,” and “fatty acid metabolic process” within biological processes, reflecting mid-vein vasculature’s role in cell membrane dynamics and transport. Cellular component classification highlighted connections with “cell wall,” “external encapsulating structure,” “apoplast,” “cell periphery,” and “extracellular region,” indicating mid-vein vasculature’s involvement in sucrose modulation and cell osmotic equilibrium. In the realm of molecular function, DEGs were linked to “tethering complex,” “terpene synthase activity,” “carbon-oxygen lyase activity,” “heme binding,” and “tetrapyrrole binding,” suggesting mid-vein vasculature’s engagement in hormone and sucrose transduction.

**Figure 5:**
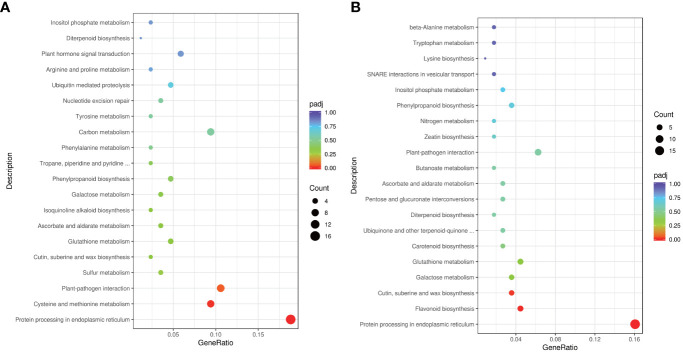
KEGG term for HL **(A)** and HV **(B)**.

To delve deeper into gene functions, we conducted KEGG pathway classification (**Figure 5**). The three most enriched pathways in HL were “Protein processing in endoplasmic reticulum,” “Cysteine and methionine metabolism,” and “Plant-pathogen interaction” (**Figure 5A**). Conversely, the top three enriched KEGG pathways in HV encompassed “Protein processing in endoplasmic reticulum,” “Flavonoid biosynthesis,” and “Cutin, suberine, and wax biosynthesis” (**Figure 5B**), further highlighting distinct pathway involvement between HV and HL. In sum, our findings underscore the variances in heat stress-responsive molecular activities between cassava mid-vein vasculature and leaf mesophyll. Such insights broaden our understanding of tissue-specific responses to heat stress and hold promise for advancing heat tolerance strategies in crops.

### Validation of RNA-seq gene expression via QPCR

**Figure 6:**
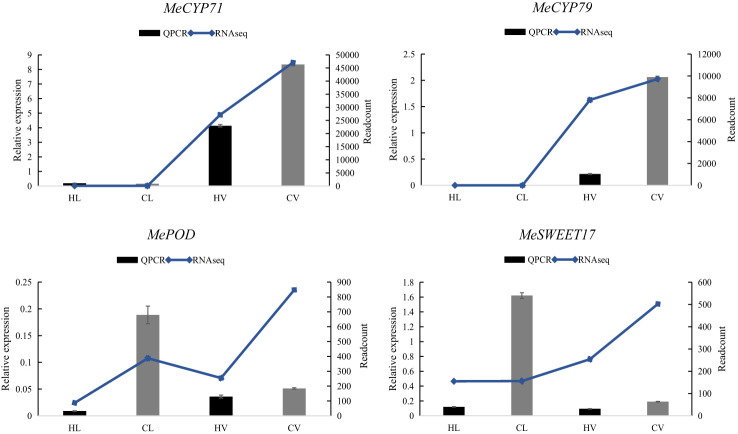
Relative expression of four representative genes in HT stressed KU50 leaf and mid-vein. QPCR relative expression corresponds to log2 fold change of the ΔΔCT values normalized with the actin gene. Each column represents the mean value plus standard deviation from three biological replicates.

To validate the RNA-Seq expression data, we conducted QPCR on four random DEGs, *MeCYP71*, *MeCYP79*, *MePOD*, *MeSWEET11*. Our assessment demonstrated a consistent correlation between the RNA-Seq and QPCR results, despite variations in the fold change magnitudes of gene expressions (**Figure 6**). Among the selected genes, *MeCYP71* and *MeCYP79* are key players in cyanogenic glucoside biosynthesis. In this process, CYP79 enzymes catalyze the conversion of amino acids to oximes, crucial for cyanogenic glucoside synthesis. This phenomenon is well-documented across several plant species, including cassava. Notably, knockout of CYP79D1 has been shown to substantially reduce linamarin and cyanide levels in leaves, emphasizing their importance (Jorgensen et al., 2011; Juma et al., 2022). Our results consistently highlighted higher enrichment of *MeCYP71* and *MeCYP79* in mid-vein vasculature compared to leaf mesophyll, corroborated by both RNA-Seq and QPCR data. Notably, certain CYPs have been localized in the vasculature of Arabidopsis, while *MeCYP71E* was specifically identified in the cortex cells of cassava’s vascular tissue (Bak et al., 2011; Jorgensen et al., 2011). Another gene, *MePOD*, associated with stress responses and postharvest physiological deterioration conditions (Wu et al., 2019), exhibited reduced expression under heat stress. Similarly, *MeSWEET11*, a transporter implicated in sugar transport through diverse conformational states during the transport cycle (Eom et al., 2015), displayed diminished expression under high-temperature stress conditions. These consistent trends reinforced the robustness and accuracy of our RNA-Seq findings.

### Heat shock proteins involved in all tissue response to the HT stress

**Figure 7:**
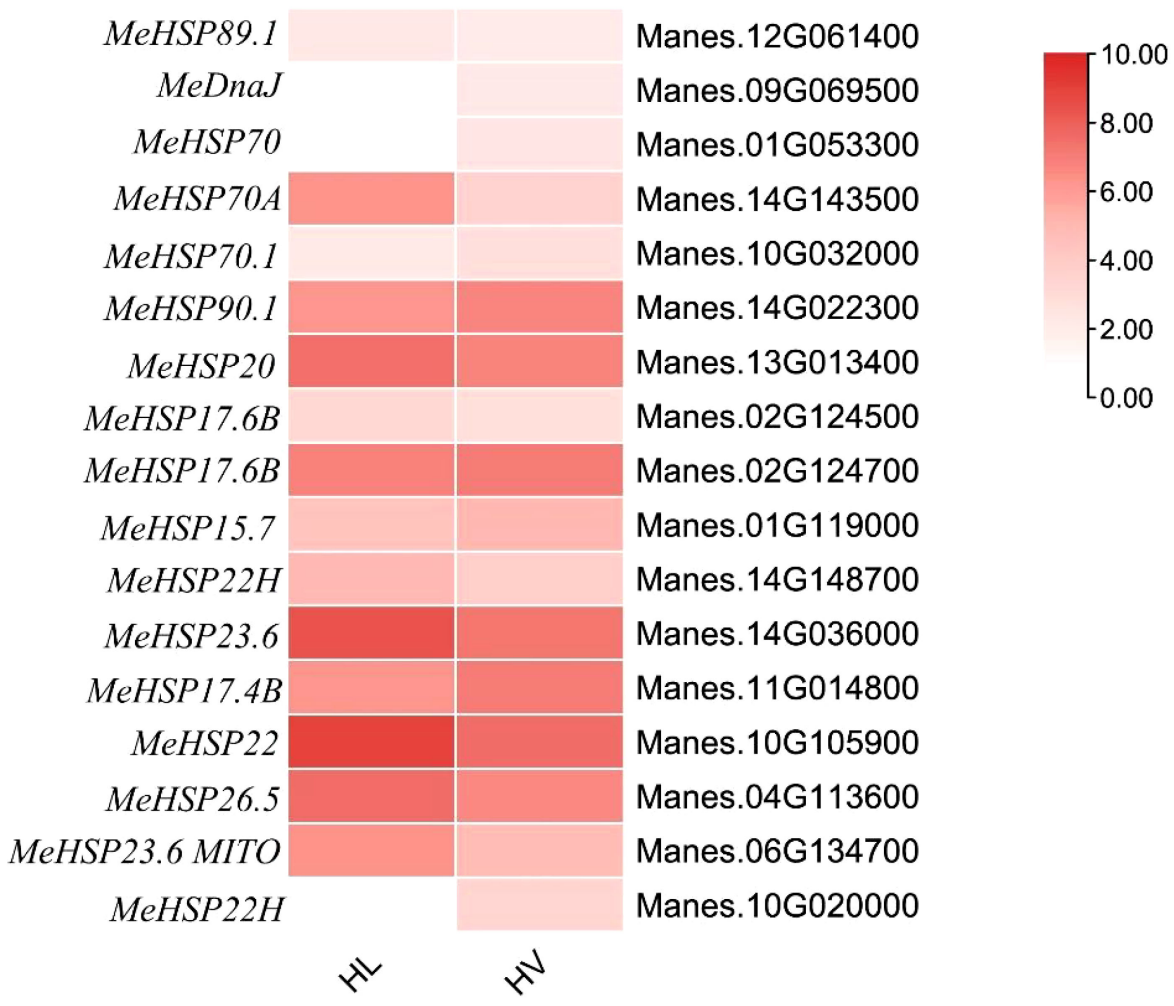
Heat map for HSPs’ expression in KU50 leaf mesophyll and vascular bundle under HT stress (HL, HT stressed leaf mesophyll; HV, HT stressed vein. Red bar represents foldchange.).

In plants, Heat shock proteins (HSPs) are induced by stress and are proposed to act as molecular chaperones to protect other proteins from stress-induced damage (Waters and Vierling, 2020). Notably, HSPs play a pivotal role in orchestrating gene expression alterations that enable plant survival under HT conditions. Furthermore, Heat Shock Factors (HSFs) can also modulate HSPs’ activity, contributing to plant adaptation to HT stress (Khan and Shahwar, 2020). In our results, there are many HSPs and HSFs had been increased significantly in both leaf mesophyll tissue and mid-vein (**Figure 7**). Intriguingly, among these genes, only three were exclusively expressed within the vascular bundle, distinct from their expression in leaf mesophyll. These genes encompass *MesHSF* (small heat shock factor, Manes.10G020000), *MeHSP40* (Manes.09G069500), and *MeHSP70* luminal-binding protein (BiP, Manes.01G05330). These elements, alongside UDP-glucose glycoprotein glucosyl transferase, are integral components of the endoplasmic reticulum quality control (ER QC) machinery. This machinery diligently monitors the accurate folding and processing of membrane and secretory proteins(Park and Seo, 2015). Our findings underscored the induction of 17 HSPs by HT stress across the entire cassava leaf. Notably, within the cassava vascular bundle, three HSPs exhibited heightened enrichment, suggesting a distinctive responsive mechanism in cassava’s vascular system to HT stress. Furthermore, several of these HSPs demonstrated pronounced GUS staining in the vasculature, exemplified by *AtHSP90-2* (Kozeko and Kordyum, 2023).

### Transcription factors involved in the HT response of KU50

Transcription Factors (TFs), including WRKY, MYB, NAC, and AP2/ERF, are pivotal regulators of genes associated with diverse stresses, making them prime candidates for enhancing plant resistance against various stressors (Wang et al., 2016). Among these, the WRKY transcription factors hold significant importance in conferring HT resistance in plants. Notably, potato genes *StWRKY16*, *StWRKY45*, and *StWRKY55* have been identified as crucial players enabling plants to combat high-temperature stress(Gong et al., 2015). Certain WRKYs, such as *BnWRKY41* and *CsWRKY13*, play roles in regulating anthocyanin and lignin biosynthesis, respectively (Duan et al., 2018; Teng et al., 2021). Meanwhile, the MYB transcription factors exert substantial control over genes involved in anthocyanin production. Notably, the R2R3-MYB subfamily has been extensively studied for their role in anthocyanin regulation, with examples spanning various plant species. Across different plant species, numerous MYB genes have been identified as regulators of anthocyanin biosynthesis. In Arabidopsis thaliana, pivotal MYB transcription factors in anthocyanin regulation include PAP1 (PRODUCTION OF ANTHOCYANIN PIGMENT 1), MYB75, PAP2 (PRODUCTION OF ANTHOCYANIN PIGMENT 2), MYB90, and MYB113. In maize (Zea mays), several MYB transcription factors, such as C1, Pl1, and B-Peru, are associated with anthocyanin biosynthesis. The petunia (Petunia hybrida) *PhAN2* (ANTHOCYANIN2) MYB transcription factor is a key player in anthocyanin regulation. Additionally, in grapevine (Vitis vinifera), MYB transcription factors *VvMYBA1* and *VvMYBA2* are linked to anthocyanin accumulation.

**Figure 8:**
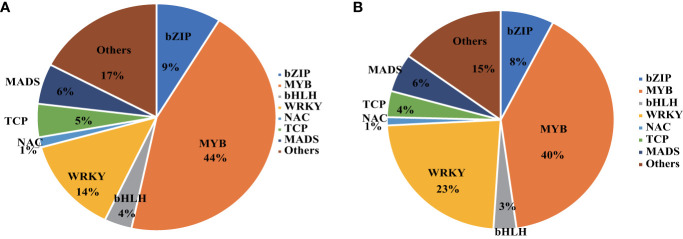
Transcription Factors enriched in KU50 leaf mesophyll **(A)** and KU50 vasculature **(B)** under the HT stress.

Our investigation yielded intriguing insights. Specifically, KU50 vasculature exhibited 829 TFs, while leaf mesophyll displayed 697 TFs under HT conditions. Remarkably, a majority of these TFs belonged to the MYB and WRKY families (**Figure 8**). Within this repertoire, numerous MYBs were implicated in the anthocyanin biosynthesis pathway, including *LIMYB3* and *StMYB44*, which contribute to anthocyanin regulation under abiotic stress conditions. Furthermore, it’s noteworthy that *LIMYB3* demonstrated expression in the Arabidopsis vasculature, further highlighting the significance of these regulators in plant stress responses (Liu et al., 2019; Yong et al., 2019).

### KU50 leaf mesophyll decreased the photosynthesis and increased the pyruvate pathway to response the HT stress

**Figure 9:**
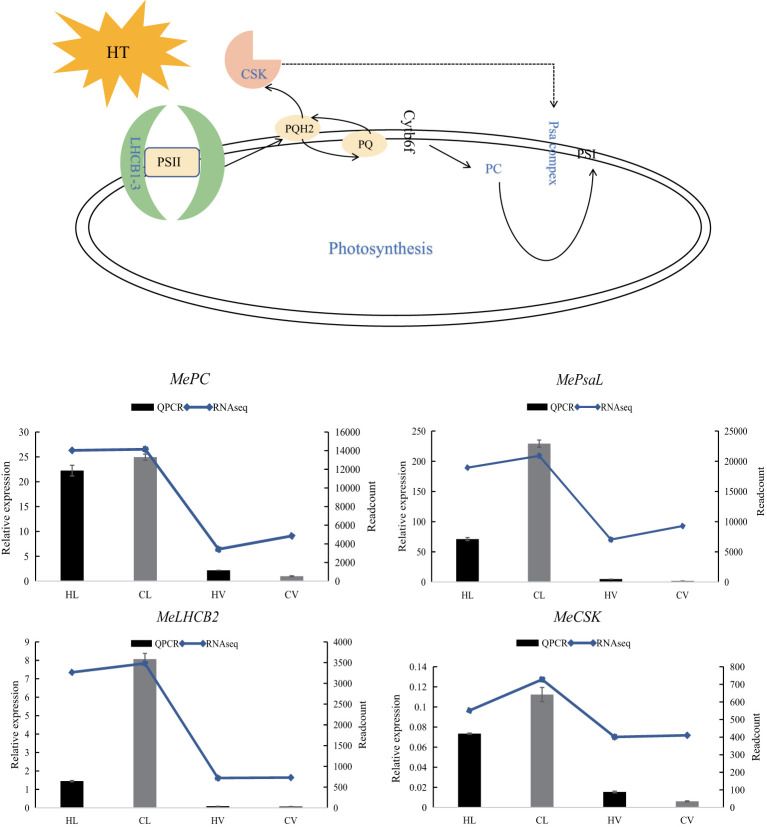
Relative expression of four representative genes in HT stressed KU50 leaf and vein. QPCR relative expression corresponds to log2 fold change of the ΔCT values normalized with the actin gene. Each column represents the mean value plus standard deviation from three biological replicates.

The adverse impact of HT stress on photosynthesis is well-documented, leading to compromised crop growth and production (Lipiec et al., 2013). Our physiological investigations affirmed this phenomenon, revealing a reduction in cassava photosynthesis following high-temperature treatment (**Figure 2**). Moreover, congruence between our transcriptome data and QPCR results with the physiological outcomes (**Figure 9**) attests to the reliability of our transcriptomic findings, confirming the presence of HT stress in cassava.

Plastocyanin (PC) is an indispensable and abundant copper (Cu) protein essential for photosynthesis in higher plants. Severe copper deficiency can disrupt photosynthetic electron transport by causing a deficiency in PC (Shahbaz et al., 2015). A specialized chloroplast sensor kinase (CSK) exists within chloroplasts, the photosynthetic organelles of plants and algae. CSK governs the transcription of chloroplast genes in response to fluctuations in photosynthetic electron transport, playing a critical role (Ibrahim et al., 2016). Integral to the light reaction pathway are Lhcb1, Lhcb2, and Lhcb3, components of the light-harvesting complex (LHC) II trimers, as well as the PsaL subunit vital for photosystem I trimer formation (Chen et al., 2020). Notably, these genes, encompassing LHCB, CSK, PC, and PsaL, function within the light reaction pathway (Jonwal et al., 2022). Intriguingly, under HT stress, these genes were predominantly downregulated, particularly in leaf tissues.

LHCBs (Light Harvest Chlorophyll Binding Proteins) serve as key players in guard cell signaling triggered by abscisic acid (ABA), suggesting their potential involvement in ABA signaling through modulation of ROS homeostasis (Xu et al., 2012). In drought-treated jatropha leaves, genes like *JcLhcb1.1*, *JcLhcb1.2*, *JcLhcb3*, *JcELIP*, *JcSEP2*, and *JcSEP5* were upregulated, underlining their role in response to water stress (Zhao et al., 2020). Similarly, *MeLHCB* demonstrated significant upregulation in KU50 leaf mesophyll under HT stress.

**Figure 10:**
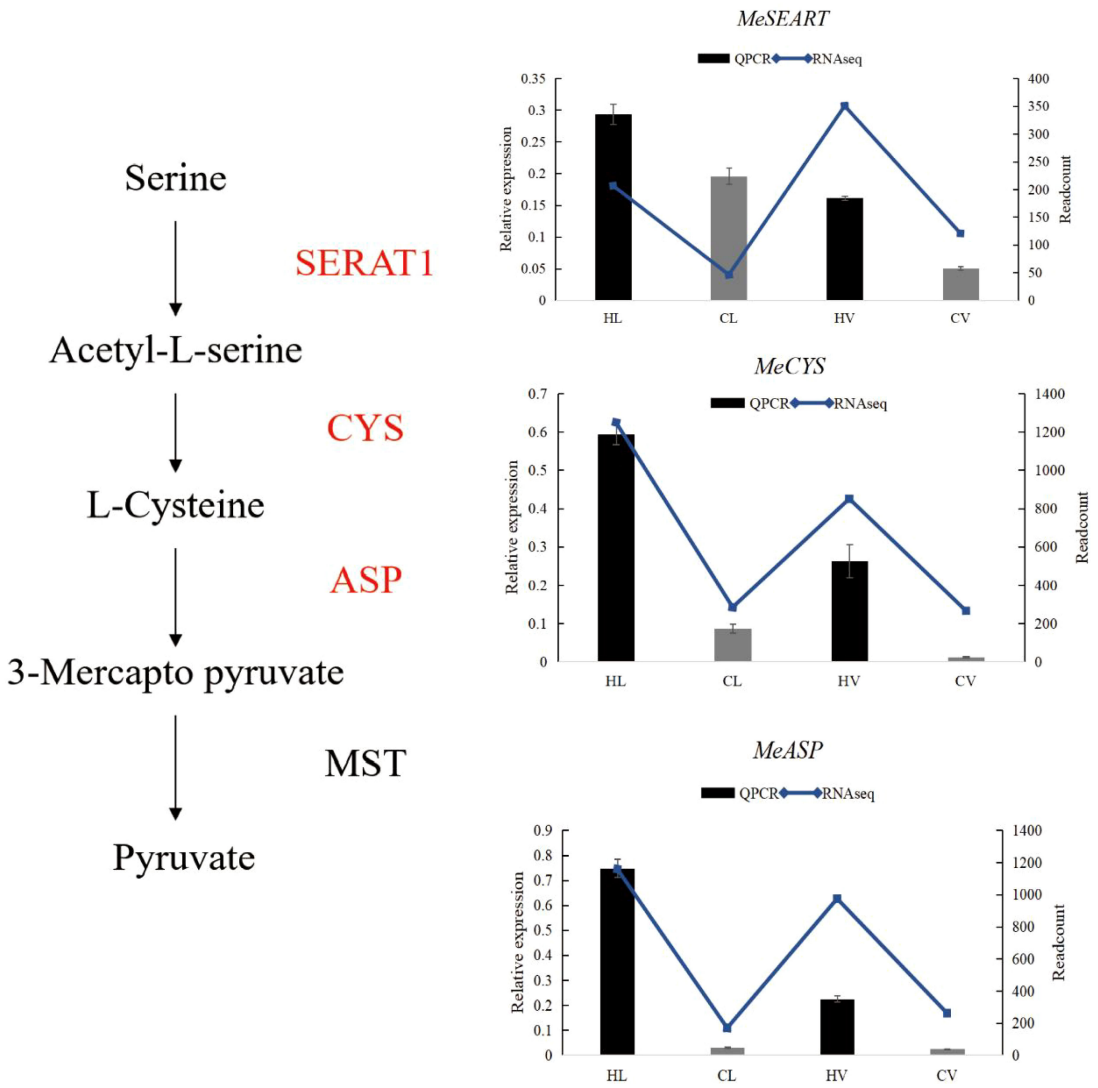
Pyruvate biosynthesis pathway in HT treated KU50 leaf mesophyll (Red color means increased expression). Relative expression of three representative genes in HT stressed KU50 leaf and vein. QPCR relative expression corresponds to log2 fold change of the ΔCT values normalized with the actin gene. Each column represents the mean value plus standard deviation from three biological replicates. SERAT1, Serine Acetyltransferase 1; CYS, Cysteine synthase; ASP, Aspartate aminotransferase. Blue line means RNA-seq readcount, column means QPCR relative expression.

Following the meticulous identification of DEGs specific to leaf mesophyll response, we meticulously classified these genes and observed a significant association with the pyruvate pathway. Pyruvate, a pivotal molecule, plays a vanguard role in neutralizing heat-induced ROS. Our findings unveil a noteworthy elevation in the expression of key genes within the pyruvate synthesis pathway under HT stress. This includes serine acetyltransferases (SERATs), cysteine synthase (CYS), and aspartate aminotransferase (ASP), with QPCR validation corroborating our RNA-seq data (**Figure 10**).

The gene family of SERATs orchestrates an essential interface between the plant’s serine and sulfur metabolic pathways. Functionally, SERATs provide the activated precursor, O-acetylserine, which underpins the incorporation of reduced sulfur into cysteine. This occurs via the exchange of the serine hydroxyl moiety with a sulfhydryl moiety, resulting in the formation of diverse organic compounds containing reduced sulfur moieties (Watanabe et al., 2018). In line with this, our results unequivocally indicate a surge in SERAT expression triggered by HT stress. Exploring the impact of enhanced expression, overexpressing *MsCYS* in Alfalfa has been shown to bolster alkali tolerance. This augmentation is attributed to the regulatory influence of *MsCYS* on osmoregulatory substances and the enhancement of antioxidant capacity (Yuan et al., 2021). Collectively, this insight underscores the potential of CYS as a key player in stress responses, with cascading benefits for improved stress tolerance. Another integral player, aspartate aminotransferase, occupies a pivotal position in the intricate orchestration of plant carbon and nitrogen allocation. Beyond this role, ASP’s influence extends to impacting pathogen defense mechanisms, thereby conferring a dynamic and multifaceted role within plant biology (Brauc et al., 2011).

### KU50 vasculature bundle altered the lignin synthesis to response the HT stress

**Figure 11:**
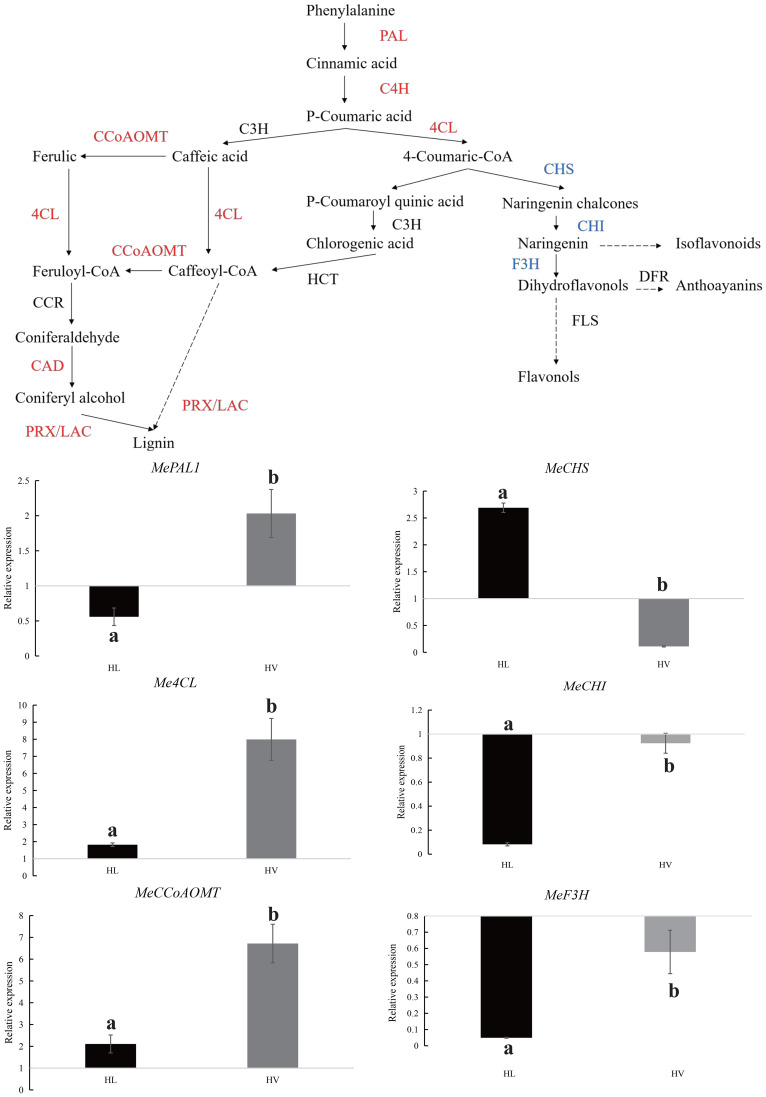
Phenylalanine metabolism pathway in HT treated KU50 mid-vein vasculature (Red color means increased expression and the blue color means decreased expression). Relative expression of four representative genes in HT stressed KU50 leaf and vein. QPCR relative expression corresponds to log2 fold change of the ΔΔCT values normalized with the actin gene. Each column represents the mean value plus standard deviation from three biological replicates.

Following the meticulous filtration of vasculature bundle-specific response DEGs, a comprehensive classification of these genes was performed, revealing a predominant association with lignin biosynthesis. A cornerstone of numerous biologically and economically significant secondary metabolites, phenylalanine synthesis underpins the accumulation of these crucial compounds. Our study unearthed a remarkable upsurge in the phenylalanine metabolism pathway within KU50 mid-vein vasculature, under HT conditions. This shift was underscored by a decrease in genes linked to flavonol synthesis, coupled with an increase in genes steering the lignin synthesis pathway (**Figure 11**). Our meticulous QPCR validation further corroborates these findings, consolidating the premise that KU50 augments its phenylalanine metabolism pathway to enhance lignin synthesis, thereby navigating the challenges posed by HT stress.

### *In situ* localization validates vascular-specific gene expression in KU50

With our focus on transcriptomic analysis within cassava’s vascular tissue, a captivating question arises: are the identified genes exclusive to the vasculature? In pursuit of an answer, we embarked on *in situ* localization experiments to delve into the spatial expression patterns of these identified genes. By conducting these assays, we aimed to shed light on the precise locations of gene expression within the KU50 mid-vein vasculature. Specifically, we employed hybridization techniques to visualize the distribution of the identified caffeoyl-coenzyme A-3-O-methyltransferase (*MeCCoAOMT*) gene. In this endeavor, we meticulously sectioned KU50 mid-vein samples and conducted hybridization experiments utilizing targeted *MeCCoAOMT* probes. Our results unveiled a striking revelation: *MeCCoAOMT* expression manifested prominently within mid-vein xylem vessels, precisely during the phase of robust lignification. This localization pattern aligns seamlessly with the findings of *PhCCoAMT *(Chen et al., 2000), reinforcing the credibility of our insights and bolstering the premise that these genes indeed exhibit vascular-specific expression.

## Discussion

High temperature caused by global warming is the one of the major environmental factors limiting crop yields. With growing population, the food crisis caused by the shortage of food supplies is becoming more and more prominent. Therefore, helping crops getting strong HT resistance agronomic trait is one of the important ways to alleviate food crisis (Wahid et al., 2007). Cassava, a tropical staple crop known for its robust productivity, boasts inherent HT tolerance (El-Sharkawy, 2006). However, perhaps precisely because it is a tropical crop, there is currently limited research on the heat tolerance of cassava. Given the distinct physiological roles of various plant tissues, their tissue-specific responses to abiotic stress come to the forefront. The vascular system plays a crucial role in plant development and stress resistance, serving functions such as mechanical support and signal transmission. Despite this, scant attention has been devoted to deciphering cassava’s HT responses, especially within its vascular tissue. Accordingly, unraveling the HT tolerance mechanisms across different cassava tissues assumes paramount importance, holding the potential to guide heat-resilient crop breeding.

In this experiment, to confirm that the primary tissue in cassava leaf mid-vein is vascular bundle, we employed toluidine blue staining on cassava leaf mid-vein, and the results confirmed that the primary component of cassava leaf mid-veins was vascular bundle. Subjecting cassava to HT treatment was further validated through thermal imaging, substantiating its exposure to HT stress. Concurrently, assessment of photosynthetic efficiency indicated a reduction under HT conditions, and there were more HSPs which were HT stress marker genes had been found increased in the HT treated groups. These multifaceted observations collectively validate the effective imposition of HT stress on cassava, a fact amply supported by our congruent QPCR results.

Subsequent to HT stress, both leaves and main veins were meticulously collected, ensuring three biological replicates for both control and treatment groups. Leveraging transcriptome sequencing, we sought to minimize errors arising from manual dissection and the residual presence of mesophyll tissue within main veins. Our meticulous approach encompassed distinct comparisons and enrichments of leaf mesophyll-specific DEGs, alongside mid-vein vasculature-specific DEGs. Intriguingly, the Venn diagram delineated 65 DEGs exclusively responding to HT within cassava mesophyll. Notably, some of these DEGs had previously been identified in leaf mesophyll, exemplified by Glutathione S-transferases (GSTs), a protein family pivotal in oxidative damage mitigation (Kumar and Trivedi, 2018), as well as *SbGST* found within leaves (Tiwari et al., 2016). Likewise, among the 93 DEGs specific to vascular bundles, select genes including *Os4CL* had been validated for their vasculature location in rice (Gui et al., 2011). A subset of four randomly selected genes underwent QPCR validation, with the resulting trends mirroring the transcriptome data, underscoring the reliability of our findings. The outcomes collectively suggest a heightened responsiveness of cassava leaf vasculature to HT stress, a differential behavior expounded by distinct molecular mechanisms unveiled through GO and KEGG analyses.

### Cassava mesophyll reduced the damage of peroxides under HT by improving pyruvate synthesis

Photosynthesis, a pivotal physiological process, is acutely sensitive to heat stress. Particularly, the activity of photosystem II (PSII) can be significantly reduced or even halted under HT conditions, owing to the heightened vulnerability of the PSII complex [51]. The comprehensive analysis of transcriptome data unveiled the down-regulation of photosynthesis-related genes in cassava leaves, aligning harmoniously with corroborative QPCR results and physiological assessments of photosynthetic activity. Of intrigue is the observation that despite HT causing a decrease in cassava’s stomatal conductance and related physiological markers, the intercellular CO_2_ concentration remained relatively stable. This curious outcome may be attributed to cassava’s dual C3 and C4 facultative crop characteristics. This is because cassava closes its stomata in water-deficient conditions, yet it can still recycling CO_2_ through the phosphoenolpyruvate to resist drought (Punyasu et al., 2023).

Concurrently, our investigation shed light on the pyruvate synthesis pathway, which exhibited a notable up-regulation in mesophyll tissues. This phenomenon suggests an augmented pyruvate content within the mesophyll. This is because research has indicated that under abiotic stress, pyruvate can mitigate the damage caused by peroxides to plants. For example, in celery, increasing pyruvate content through exogenous melatonin enhances its heat tolerance (Li et al., 2022). Our results are consistent with previous research findings, indicating that cassava leaves respond to HT stress by increasing pyruvate content. In summary, we hypothesize that the heat tolerance mechanism in cassava leaves may involve mitigating damage caused by HT through enhanced pyruvate metabolism.

### The vascular bundles of cassava leaves resist HT by enhancing lignin synthesis

**Figure 12:**
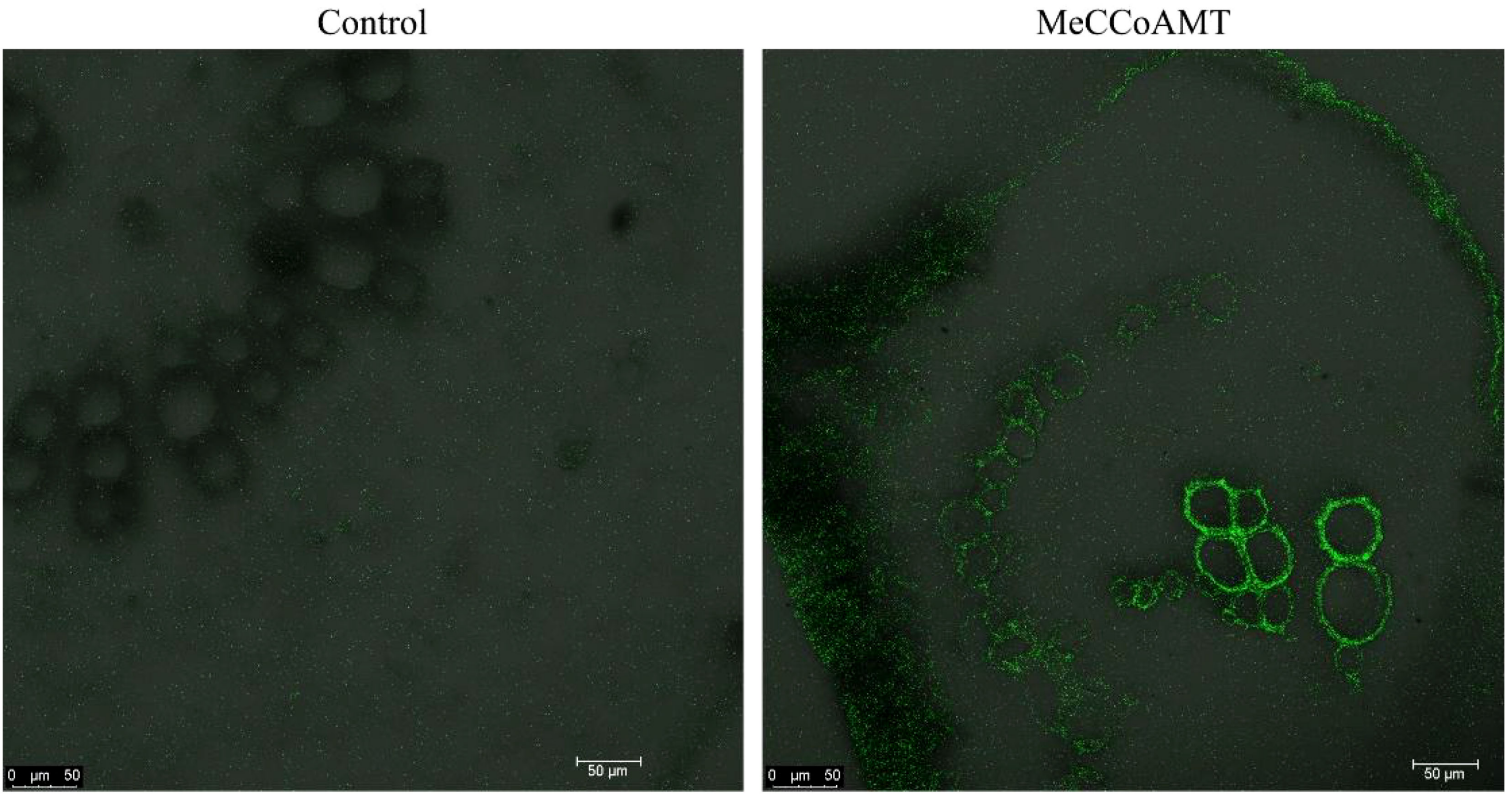
*In situ* location of *MeCCoAOMT* in transversal sections of KU50 mid-vein by microscopy.

Plants tend to synthesize more lignin to enhance their HT resistance. Because during lignification, the deposition of lignin in the cell wall enhances its stiffness and reduces the permeability of the xylem cell wall to water. This facilitates the efficient long-distance transport of water, minerals, and organic matter within the plant. For instance, poplar increased the lignin biosynthesis in stem to cope with HT stress (Zhao et al., 2022). In the transcriptome results of vascular bundle, phenylalanine metabolism pathway was significantly enriched in the HT treated group. Phenylalanine, a precursor to crucial metabolites, serves as the foundation for both lignin and anthocyanin synthesis. Our findings showcased an evident up-regulation of the phenylalanine metabolic pathway, alongside a concurrent down-regulation of genes linked to the downstream anthocyanin synthesis pathway, and an up-regulation of corresponding lignin synthesis pathway genes. This study was further substantiated through QPCR verification of pivotal genes from the phenylalanine, anthocyanin, and lignin pathways, with results harmonizing with transcriptome trends. These combined results signify the predominant response of cassava’s leaf mid-vein vascular bundle to HT stress via the orchestrated regulation of lignin synthesis. Additionally, *in situ* hybridization assays targeting *MeCCoAMT*, a pivotal gene in the lignin biosynthesis pathway implicated in stress tolerance and disease resistance (Lam et al., 2007), revealed its expression within the xylem of cassava leaf mid-vein (**Figure 12**).

The integral role of lignin in plant cell walls and its malleability in response to changing environments is well acknowledged (Endler et al., 2015). The cell wall often acts as a frontline defense against stressors and intrusions (Van Sandt et al., 2007). Similar to our findings, the expression of similar genes was observed in coffee leaves under HT stress, reflecting the modification of cell wall structure and composition (Lima et al., 2013). The similar genes’ expression had been found in our results (**Table 2**). HT-induced expression of *MeXET* (Xyloglucan Endotransglucosylase) in cassava’s vascular bundle underlines its direct involvement in shaping and restructuring plant cell walls (Fry, 2004). The wheat Lipid Transfer Protein 3 (*TaLTP3*), when overexpressed in Arabidopsis, confers enhanced thermotolerance at the seedling stage (Wang F. et al., 2014). Similarly, its counterpart in cassava (*MeLTP*) exhibited heightened expression under heat stress in our study. Augmented expression of *MeWAK* (Wall-associated receptor kinase), a receptor for pectins, associated with cell expansion during plant development (Kohorn and Kohorn, 2012), further corroborates vascular bundle adaptation to HT stress. Moreover, the down-regulation of *MeGH* (glycoside hydrolases), which catalyze the hydrolysis of plant cell wall polysaccharides (Su et al., 2012), provides insights into how cassava’s vascular bundles strategically enhance lignin biosynthesis to fortify cell wall integrity, effectively responding to HT stress.

**Table 2:** Vasculature specific DEGs in HT treated KU50.

Gene ID	Gene name	Log2 foldchange	p-Value
Manes.14G114400	XET	2.33	0.0003
Manes.10G152900	lipid transfer	2.29	0.0005
Manes.06G000800	Pyridoxal-dependent decarboxylase	2.21	1.6993346092335E-13
Manes.12G104300	O-methyltransferase	2.13	0.0028
Manes.04G099400	Wall-associated receptor kinase	2.09	0.00014
Manes.12G006700	Sugar efflux transporter	2.05	0.0002
Manes.12G144000	P450	-2.97	0.0002
Manes.12G144200	P450	-2.86	0.00004
Manes.10G037200	Glutathione S-transferase	-2.81	8.59232545070319E-19
Manes.10G144100	Glycosyl hydrolase	-2.55	5.8010244088709E-12
Manes.11G072700	Glycosyl hydrolase	-2.54	2.750764917365E-10

## Conclusions

In most studies on plant HT tolerance, there has been limited classification research distinguishing between leaf mesophyll and leaf vein vascular bundles. Instead, leaves are often studied as an entity, potentially causing us to overlook crucial information. This is significant because under HT stress, signals generated within plant leaf tissues rely on vascular bundles for transmission. Furthermore, different tissues should possess distinct adaptive mechanisms to environmental stress, and vascular bundles require their own defenses against HT. Our experimental results demonstrate that leaf vein vascular bundles and mesophyll exhibit distinct response mechanisms under HT conditions. Through differential expression analysis, we identified genes within both cassava leaf mesophyll and mid-vein vascular tissue that dynamically responded to the challenges imposed by HT stress. Intriguingly, our investigation revealed substantial disparities in the transcriptomic profiles of these distinct tissue types. We propose a compelling hypothesis that within the same leaf, the mid-vein vascular tissue activates distinct responsive mechanisms in contrast to the leaf mesophyll when confronted with HT stress. This suggests an intricate tissue-specific orchestration of adaptive strategies under these conditions. Specifically, while leaf mesophyll tissue experiences a decline in photosynthesis rates due to HT stress, it concurrently activates an up-regulated pyruvate synthesis pathway to counteract the thermal stress. In parallel, the mid-vein vascular tissue notably enhances the phenylalanine metabolism pathway, promoting the synthesis of lignin to reinforce its resilience against HT stress. These findings represent a significant advancement in comprehending how different tissues within cassava modulate gene expression patterns in response to HT stress. Our study on the heat tolerance mechanisms of cassava can offer valuable insights for heat-resistant breeding in a broader range of crops and provide reference points for the expansion of temperate crops to more southern regions.

## Data availability statement

The raw sequence data reported in this paper have been deposited in the Genome Sequence Archive (Genomics, Proteomics & Bioinformatics 2021) in National Genomics Data Center (Nucleic Acids Res 2022), China National Center for Bioinformation / Beijing Institute of Genomics, Chinese Academy of Sciences (GSA: CRA010323) that are publicly accessible at https://ngdc.cncb.ac.cn/gsa.

## Author contributions

SW: Conceptualization, Data curation, Formal analysis, Investigation, Methodology, Project administration, Software, Supervision, Validation, Visualization, Writing – original draft, Writing – review & editing. XZ: Writing – review & editing. KP: Methodology, Software, Writing – review & editing. HZ: Software, Methodology, Writing – review & editing. XS: Methodology, Software, Writing – review & editing. JL: Software, Writing – review & editing. YL: Writing – review & editing, Software. YC: Writing – review & editing. WW: Writing – review & editing, Funding acquisition, Resources, Supervision, Visualization, Writing – original draft.
