# Supplementary figures and images for: Distinct heat response molecular mechanisms emerge in cassava vasculature compared to leaf mesophyll tissue under high temperature stress
Source: Front Plant Sci. 2023 Nov 30;14:1281436. doi: 10.3389/fpls.2023.1281436 (PMC10720452; doi:10.3389/fpls.2023.1281436)

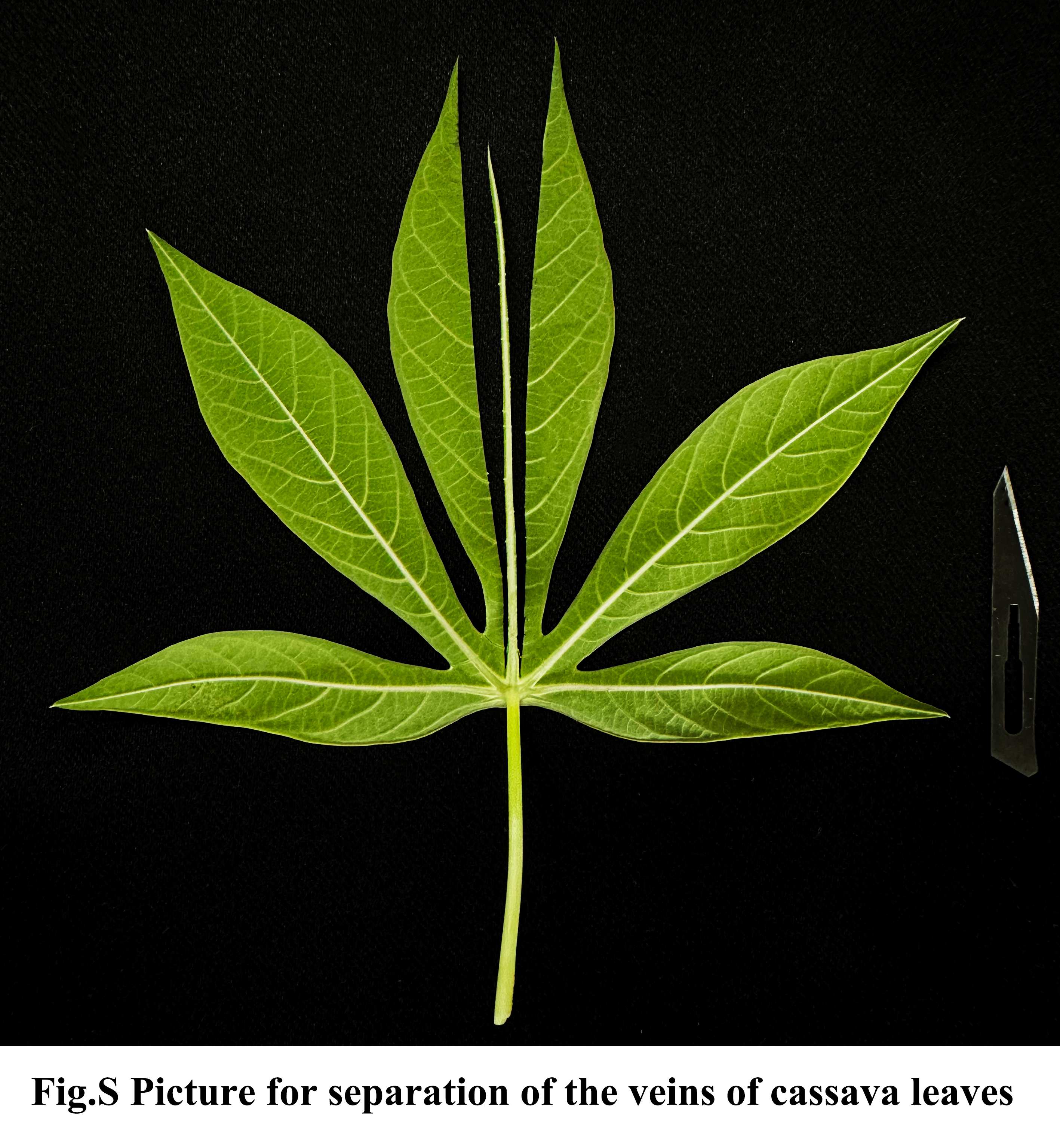

Supplement: Supplementary file 1 [file Image_1.jpeg]
